# Molecular mechanism for pancreatic β-cell dysfunction and atherosclerosis

**DOI:** 10.1007/s13340-025-00871-5

**Published:** 2026-01-21

**Authors:** Hideaki Kaneto

**Affiliations:** Department of Diabetes, Endocrinology and Metabolism, Kawasaki Medical School, 577 Matsushima, Kurashiki, 701-0192 Japan

**Keywords:** Pancreatic β-cell dysfunction, Atherosclerosis,, Oxidative stress, Incretin, Imeglimin

## Abstract

It is well known in clinical practice that when pancreatic β-cells are chronically exposed to hyperglycemia, β-cell function is gradually deteriorated. It has been revealed that under diabetic conditions oxidative stress is provoked and expression levels of insulin gene transcription factors and incretin receptors are down-regulated which are closely associated with β-cell glucose toxicity. We showed that expression levels of these factors were preserved by reducing glucose toxicity with SGLT2 inhibitor. In addition, we showed that it was more beneficial to use incretin-based drugs at an early stage of diabetes when incretin receptor expression was preserved in β-cells. Similarly, we showed that expression levels of incretin receptors in arterial cells were down-regulated which seemed to be associated with the progression of atherosclerosis. Imeglimin is a relatively new anti-diabetic drug and has been used in clinical practice. Recently we have reported that imeglimin exerts beneficial effects on mitochondria morphology in β-cells and/or number and quality of insulin granules. In addition, we have reported that imeglimin shows favorable effects against the development of atherosclerosis independently of glycemic and lipid control. Taken together, it is likely that augmentation of oxidative stress and decreased expression levels of insulin gene transcription factors and incretin receptors are closely associated with pancreatic β-cell glucose toxicity. In addition, incretin-based drugs and imeglimin are expected to exert favorable effects against β-cell glucose toxicity and the development of atherosclerosis when they are appropriately introduced.

## Clarification of molecular mechanism for pancreatic β-cell glucose toxicity: involvement of oxidative stress and suppression of insulin gene transcription factors PDX-1 and MafA

In 1992, I started basic research as a graduate student in Department of Biochemistry in Osaka University under the supervision of Prof. Naoyuki Taniguchi. The main theme at that time was “involvement of oxidative stress in diabetes mellitus”. For example, we proposed the following working hypotheses. (a) Nitric oxide induces apoptotic cell death in pancreatic β-cells which seems to lead to the onset of type 1 diabetes mellitus. (b) Oxidative stress is involved in pancreatic β-cell glucose toxicity which is often observed in type 2 diabetes mellitus in clinical practice.

**Fig. 1:**
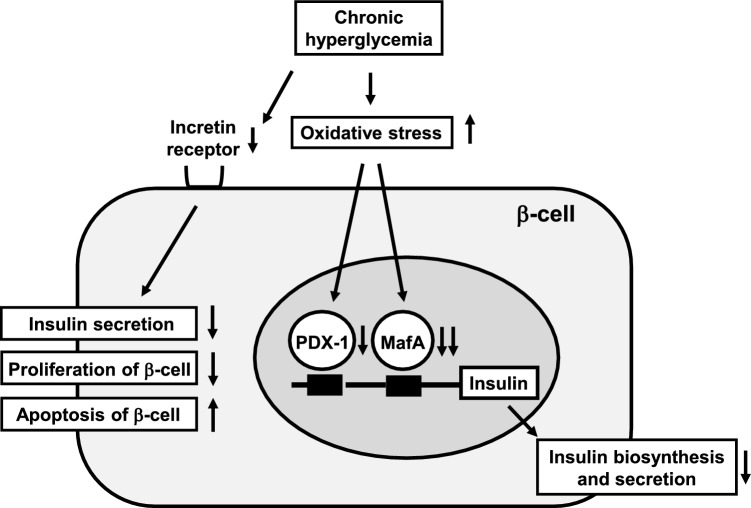
Molecular mechanism for pancreatic β-cell glucose toxicity. Under diabetic conditions, pancreatic β-cell function is gradually deteriorated by burden of glucose toxicity. It has been revealed that expression levels of insulin gene transcription factors MafA and PDX-1 and incretin receptors are down-regulated which seems to be closely associated with β-cell glucose toxicity. Such deterioration of β-cell function leads to vicious cycle and finally aggravates type 2 diabetes mellitus

After obtaining PhD in 1996, we continued research about “involvement of oxidative stress in type 2 diabetes mellitus” in the First Department of Medicine, Osaka University. For example, we obtained the following data. (a) Oxidative stress was provoked under diabetic conditions through the glycation reaction. (b) Pancreatic β-cells were quite vulnerable to oxidative stress due to very low expression levels of antioxidant enzymes in β-cells. (c) Expression level of PDX-1, a very important transcription factor for insulin gene, was reduced which seemed to be involved in β-cell glucose toxicity (Fig. 1).

In 1999, I continued basic research in Joslin Diabetes Center under the supervision of Prof. Gordon C Weir and Prof. Susan Bonner-Weir. We proposed the following working hypotheses based on the basic data. (a) Oxidative stress induces c-Myc expression in β-cells which is involved in β-cell glucose toxicity. (b) Oxidative stress activates the JNK pathway in β-cells which is also involved in β-cell glucose toxicity.

In 2002, I returned to Japan and continued basic research in First Department of Medicine and Department of Metabolic Medicine, Osaka University. We showed that oxidative stress and subsequent activation of the JNK pathway were involved in both pancreatic β-cell dysfunction and insulin resistance in various insulin target tissues such as the liver, adipose tissues and skeletal muscle. Furthermore, we showed that expression level of MafA, a very important insulin gene transcription factor, was markedly reduced under diabetic conditions [1–3] (Fig. 1). Furthermore, we reported that in β-cell-specific and conditional (tamoxifen-induced) MafA overexpressing transgenic db/db mice, serum insulin levels were significantly higher and blood glucose levels were significantly lower compared to their littermates [4]. These data indicate that reduction of MafA expression is closely involved in pancreatic β-cell glucose toxicity.

## Evaluation of the efficacy of reducing glucose toxicity using SGLT2 inhibitor

**Fig. 2:**
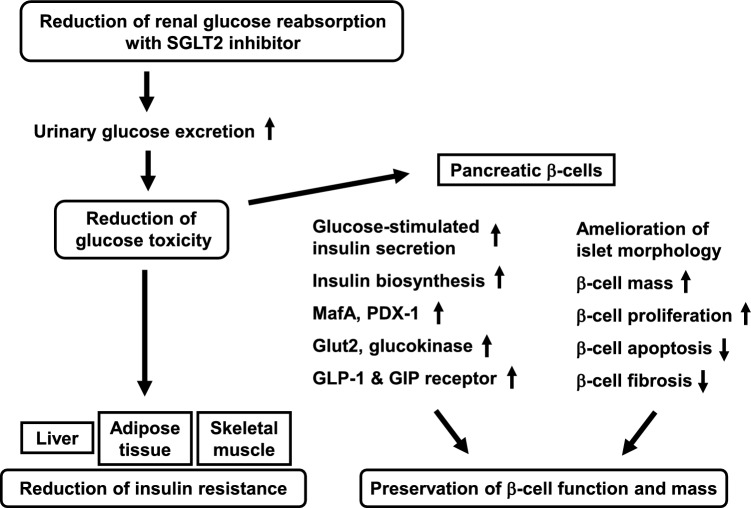
Effects of SGLT2 inhibitor on type 2 diabetes mellitus. Usage of SGLT2 inhibitor preserves pancreatic β-cell function and mass. SGLT2 inhibitor reduces β-cell glucose toxicity which leads to increase expression levels of MafA, PDX-1 and incretin receptor, and increase of insulin biosynthesis and secretion. In addition, SGLT2 inhibitor mitigates insulin resistance in various insulin target tissues such as the liver, adipose tissue and skeletal muscle

In 2014, I got transferred to Department of Diabetes, Endocrinology and Metabolism, Kawasaki Medical School and continued to perform basic research about β-cells. At the same year, SGLT2 inhibitors were launched on the market. Since SGLT2 inhibitors are thought to reduce β-cell glucose toxicity by increasing urinary glucose excretion, we evaluated the efficacy of reducing glucose toxicity using SGLT2 inhibitor. As the results, SGLT2 inhibitor increased not only MafA and PDX-1 expression and incretin receptor expression but also insulin biosynthesis, secretion and β-cell mass (Fig. 2). In addition, SGLT2 inhibitor mitigated insulin resistance in insulin target tissues. Furthermore, we showed that SGLT2 inhibitor exerted more stronger effects on insulin biosynthesis and β-cell mass when they were used at an early stage of diabetes [5].

We also showed that expression levels of MafA and PDX-1 in pancreatic β-cells were recovered by insulin therapy especially at an early stage of diabetes, which ameliorated β-cell function. In addition, we showed in clinical research that insulin therapy at an early stage of diabetes increased the possibility of withdrawal of insulin and that results in glucagon test or casual C-peptide index were useful to predict the possibility of withdrawal from insulin [6, 7].

## Screening of molecules which directly increase MafA and/or PDX-1 expression

It is known that there is insulin signaling in endothelial cells but that such insulin signaling is aggravated under diabetic conditions. Reduction of insulin signaling in endothelial cells reduces eNOS expression, which leads to reduction of blood flow and angiogenesis. Finally, pancreatic β-cells are exposed to hypoxia and/or ischemia which leads to reduction and MafA and PDX-1 expression and β-cell dysfunction [8]. Furthermore, we screened molecules which directly increase MafA and/or PDX-1 expression. Previously, we showed that MafA and PDX-1 expression level was preserved by mitigating glucose toxicity using some anti-diabetic drugs. In addition, we previously reported that preservation of MafA expression in β-cells using the Cre-loxP system led to recovery of insulin biosynthesis and secretion and finally recovery of glycemic control. Based on these experimental data, we screened molecules which directly increased MafA and/or PDX-1 expression and reported that 2 kinds of G-protein-coupled receptor compounds (fulvestrant and dexmedetomidine hydrochloride) directly increased MafA and PDX-1 expression levels [9].

## Reduction of incretin receptor expression under diabetic conditions in β-cells and arterial cells

We showed that expression levels of incretin receptors in β-cells were reduced under diabetic conditions. We think that reduction of incretin receptor expression levels in β-cells, at least in part, explain the molecular mechanism of β-cell dysfunction and indicates the importance of usage of incretin-related drug at an early stage of diabetes [10] (Fig. 1). Similarly, expression levels of incretin receptors in arterial cells are reduced under diabetic conditions [11, 12]. We think that reduction of incretin receptor expression levels in arterial cells, at least in part, explain the molecular mechanism of atherosclerosis and indicates the importance of usage of GLP-1 receptor activator at an early stage of atherosclerosis [10]. Recently, we reported that GIP/GLP-1 receptor dual activator and GLP-1 receptor activator exerted protective effects on β-cells to the similar extent, although GIP/GLP-1 receptor dual activator exerted more marked effects on fatty liver compared to GLP-1 receptor activator. We think that these data suggest that GIP signaling and GLP-1 signaling are similarly important in β-cells [14].

## Imeglimin exerts protective effects against pancreatic β-cell dysfunction and atherosclerosis

Recently, we showed the effects of imeglimin, a new anti-diabetic drug, on pancreatic β-cell function. In electron microscopic examination, imeglimin showed beneficial effects on morphology in β-cell mitochondria in type 2 diabetic mice. In addition, this drug increased the quality and number of insulin granules and decreased the percentage of apoptosis in β-cells. [15].

Furthermore, we have reported that imeglimin shows beneficial effects on the progression of atherosclerosis in STZ-induced diabetic ApoE KO mice, independently of glycolipid metabolism. Indeed, this drug decreased oxidative stress, inflammation and inflammasome in vascular cells. Finally, this drug markedly reduced expression levels of macrophage markers and the proliferation of vascular smooth muscle cells in diabetic ApoE KO mice. [16].

## Mineralocorticoid signaling is associated with the progression of atherosclerosis independently of blood pressure and glycolipid metabolism

It has been thought that mineralocorticoid (MC) receptor signaling is closely involved in the progression of atherosclerosis. Recently, we have reported that specific blocking of MC signaling with esaxerenone shows beneficial effects on the development of atherosclerosis in STZ-induced diabetic ApoE KO mice. This drug reduced expression levels of various factors related to inflammation or oxidative stress. Finally, specific blocking of MC signaling with this drug showed beneficial effects on the development of atherosclerosis, independently of blood pressure or glycolipid metabolism [17].
